# In the absence of Alzheimer’s Disease pathology, PET-measured hippocampal tau is reduced in association with greater depressive symptoms: Could tau PET be sensitive to physiological tau involved in neurogenesis?

**DOI:** 10.1016/j.jadr.2026.101071

**Published:** 2026-05-08

**Authors:** Seyed Hani Hojjati, Xiuyuan Hugh Wang, Liangdong Zhou, Samantha Keil, Emily Tanzi, Tom Maloney, Farnia Feiz, Sudhin Shah, Lidia Glodzik, Yi Li, Gloria C Chiang, Tracy A Butler

**Affiliations:** Brain Health Imaging Institute, Department of Radiology, Weill Cornell Medicine

**Keywords:** Positron emission tomography (PET), Alzheimer’s disease, Hippocampus, Depression, Neurogenesis, Amyloid, Tau

## Abstract

**Background:**

Tau Positron Emission Tomography (PET) visualizes hyperphosphorylated tau tangles which, together with Aβ plaques, are hallmark pathologies of Alzheimer’s disease (AD). Greater tau, measured by PET or neuropathology, is robustly linked to worse cognition in AD, but associations with mood are less studied. We previously reported an unexpected inverse relationship between depressive symptoms and PET-measured medial temporal tau in individuals without AD. Here, we confirm and extend this finding in an independent cohort.

**Methods:**

We analyzed cross-sectional data from 403 Alzheimer’s Disease Neuroimaging Initiative participants who underwent flortaucipir (tau) PET, Aβ PET, MRI, and clinical assessments including the Geriatric Depression Scale (GDS). Participants were categorized as Aβ− or Aβ+. Associations between GDS scores and mean hippocampal flortaucipir uptake were assessed using Spearman correlation and multiple regression.

**Results:**

In Aβ− individuals (*n* = 252), higher hippocampal tau was significantly associated with lower depression scores, whereas Aβ+ individuals (*n* = 151) showed a trend-level positive association. This difference was supported by a significant interaction between depression and Aβ status in regression analyses. Results were unchanged after adjustment for age, diagnostic group, and hippocampal volume.

**Conclusions:**

In the absence of AD pathology, greater severity of depressive symptoms is associated with lower PET-measured hippocampal tau. Because impaired hippocampal neurogenesis is considered a key mechanism in depression, and tau is a normal component of cell division including neurogenesis, this replicated inverse relationship may reflect reduced hippocampal neurogenesis. Whether tau PET can provide a window into this process in humans requires further study.

## 1 Introduction

Major depression affects approximately 280 million people worldwide and is one of the largest contributors to poor quality of life, lost productivity and healthcare costs (Herrman et al., 2022). The causes of depression are complex and involve multiple, overlapping mechanisms. One of the most intriguing but difficult-to-study mechanisms is failed neurogenesis (Eisch and Petrik, 2012; Eriksson et al., 1998; Sahay and Hen, 2007). In the 1990s it was discovered that neurogenesis occurs in adult humans, most prominently in medial temporal regions, especially the hippocampus. Reduced neurogenesis was demonstrated in animal models of depression, and many antidepressant treatments such as electroconvulsive therapy (ECT), exercise, and selective serotonin reuptake inhibitors (SSRIs) were shown to robustly boost neurogenesis. But this promising line of research has not resulted in significantly improved understanding or treatment of depression in humans. This is in large part because neurogenesis is challenging to study in humans: there are no in vivo biomarkers of neurogenesis, and attempts to image it using positron emission tomography (PET) and magnetic resonance imaging (MRI) have not proven successful (Simard et al., 2025).

Recently, in a study of 141 participants with and without AD enrolled in studies at Weill Cornel Medicine (Glaubitz et al., 2025), we suggested that PET using the radiotracer MK6240, developed to detect hyperphosphorylated tau in Alzheimer’s disease (AD), might also be sensitive to normally phosphorylated tau, which is essential for microtubule function in cell division and thus can serve as a marker of neurogenesis in preclinical studies (Wang and Mandelkow, 2016; Hong et al., 2010). This suggestion was based on the unexpected finding that reduced PET-measured tau in the medial temporal lobe was associated with greater depressive symptoms in individuals without AD pathology. This was unexpected because tau deposition in the medial temporal lobe, whether measured by PET or neuropathology at autopsy, is robustly associated with worse cognition and function in AD, (Ossenkoppele et al., 2016; Bejanin et al., 2017) and we expected a similar association with depression given close links between AD and depression via multiple biological and psychological mechanisms (Ownby et al., 2006; Saczynski et al., 2010; Green et al., 2003). Our unexpected finding of an inverse association between depression and medial temporal tau in individuals without AD pathology is potentially consistent with the idea that tau PET may be sensitive to normally-phosphorylated tau essential for neurogenesis, but requires replication. We do this here using an independent dataset: the Alzheimer’s Disease Neuroimaging Initiative (ADNI).

ADNI is a public-private partnership launched in 2003 to evaluate whether MRI, PET, other biomarkers, and clinical/neuropsychological measures can be combined to track disease progression in AD. Using the ADNI dataset, we assessed the correlation of depressive symptoms, measured using the Geriatric Depression Scale (GDS), with tau deposition in hippocampus, measured using the first-generation tau PET radiotracer flortaucapir, in individuals with and without AD pathology, defined based on Aβ status. We hypothesized that, among individuals without AD pathology (Aβ− ), greater depressive symptoms would be associated with lower hippocampal tau PET signal, potentially consistent with reduced neurogenesis. In contrast, among individuals with AD pathology (Aβ+), any neurogenesis-related tau PET signal would be overwhelmed by pathologic tau deposition, rendering such associations undetectable or reversed.

## 2. METHODS

### 2.1. Participants and clinical assessment

We included 403 ADNI participants (mean age 73.2 ± 7.4 years; 217 female) with tau-PET, Aβ-PET, structural MRI, and clinical assessments including the Geriatric Depression Scale (GDS). Participants were diagnosed as cognitively normal (CN; *n* = 133) or as having Mild Cognitive Impairment (MCI; *n* = 259) or AD (*n* = 11) using ADNI diagnostic criteria based on NINCDS–ADRDA guidelines. 252 participants were Aβ− and 151 were Aβ+ based on visual interpretation (Table 1).

### 2.2. Neuroimage processing

Flortaucipir PET scans were acquired 75–105 min post-injection as six consecutive 5-minute frames. Processing was performed using a customized PETSurfer workflow integrating FSL and FreeSurfer. All PET frames were first realigned to the initial frame and then summed to generate a single static PET volume. The summed image was intensity-normalized using cerebellar gray matter as the reference region to compute Standardized Uptake Value Ratio (SUVR). Finally, hippocampal SUVR values were extracted in PET space by resampling FreeSurfer-derived ROIs into native PET space.

Because hippocampal atrophy is associated with depression (Videbech and Ravnkilde, 2004), bilateral hippocampal volumes were calculated and normalized to total intracranial volumes to include as a covariate to account for this potential confound.

### 2.3. Statistical analyses

Analyses were performed using SPSS version 29.

Group differences between Aβ+ and Aβ− participants were assessed using *t*-tests for continuous variables and chi-square tests for categorical variables.

We assessed the association between depressive symptoms (GDS total score) and hippocampal tau PET SUVR using Spearman rank correlations, performed separately in Aβ− and Aβ+ participants. Correlation coefficients were compared using Fisher’s r-to-z transformation.

To formally test whether the association between depression and hippocampal tau differed by Aβ status, we performed multiple linear regression with hippocampal tau PET SUVR as the dependent variable and GDS score, Aβ status (Aβ+ vs Aβ− ), and their interaction as independent variables.

We additionally performed models including covariates relevant to potential confounding, including age, diagnostic group (cognitively normal, MCI, AD), and bi-hippocampal volume normalized to intracranial volume. All variables were entered simultaneously into the model.

## 3. Results

**Fig. 1.**
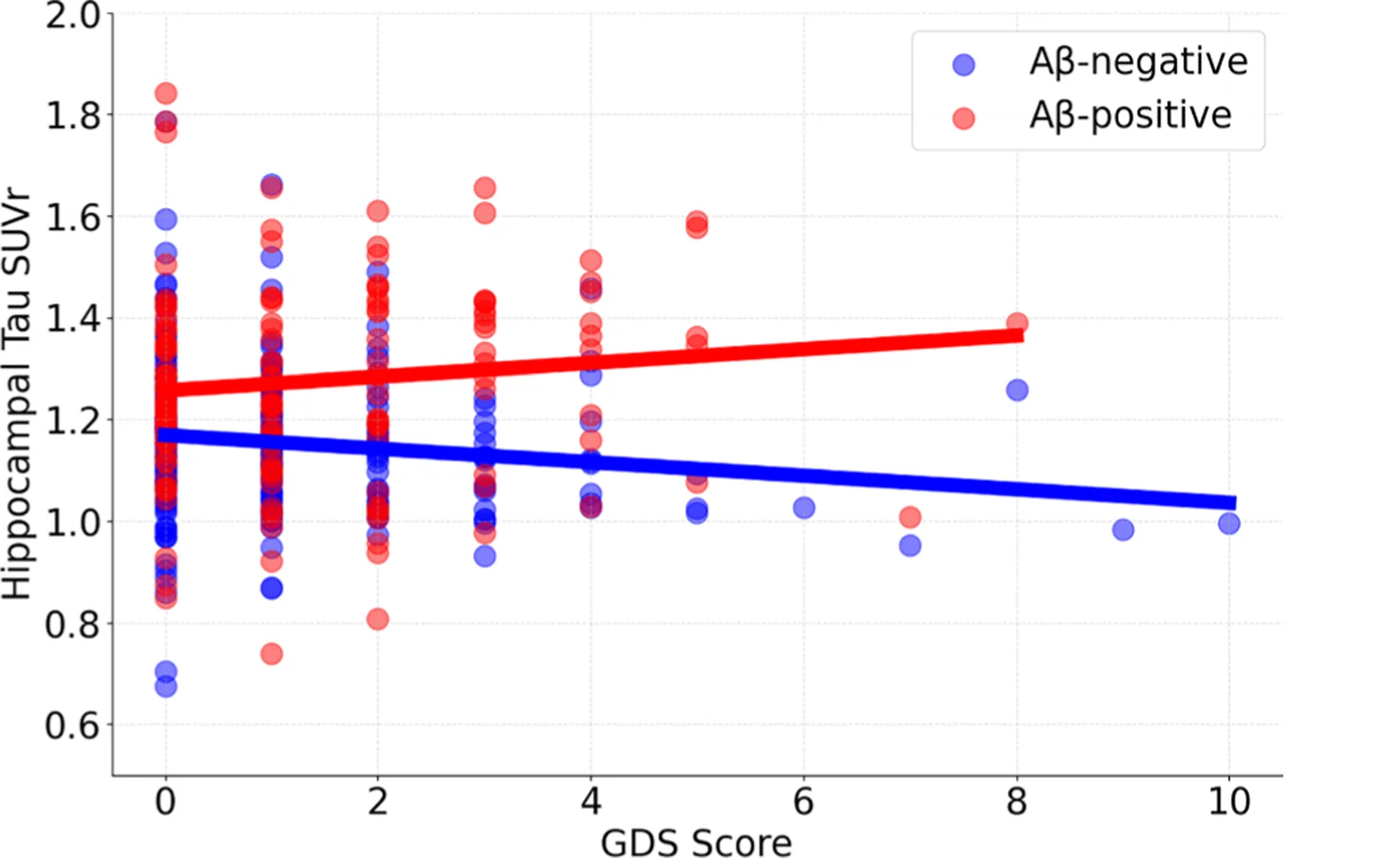
Scatter plot showing the relationship between hippocampal flortaucipir PET SUVr and Geriatric Depression Scale (GDS) score in 403 ADNI participants. In Aβ− individuals, hippocampal tau correlates inversely with depression. In Aβ+ individuals, this correlation is positive at a trend level. These divergent associations suggest distinct neurobiological mechanisms underlying depression depending on whether or not it is associated with AD pathology, with reduced hippocampal tau in individuals without AD pathology potentially reflecting reduced neurogenesis.

In Aβ− participants (*n* = 250), depressive symptoms were inversely associated with hippocampal tau (Spearman ρ = − 0.153, *p* = 0.015). In Aβ+ participants (*n* = 149), there was a trend toward a positive association (ρ = 0.146, *p* = 0.075). These correlations differed significantly (*z* = − 2.89, *p* = 0.0039). This divergent association is shown in Fig. 1.

In a regression model including GDS depression score, Aβ status, and their interaction, there was a significant interaction between depression and Aβ status (β = 0.164, *p* = 0.029), indicating that the association between depression and hippocampal tau differed by Aβ status. In this model, Aβ status was also significantly associated with hippocampal PET SUVR (β = 0.221, *p* < 0.001), whereas the main effect of depression was not significant (β = − 0.108, *p* = 0.073).

This interaction remained significant (β = 0.149, *p* = 0.046) after adjustment for age, diagnostic group, and hippocampal volume , indicating that the effect was robust to inclusion of relevant covariates. In this model, Aβ status (β = 0.224, *p* < 0.001) and diagnostic group (β = 0.145, *p* = 0.005) but not age (β = −0.062, *p* = 0.243) or hippocampal volume (β = 0.037, *p* = 0.499) were also associated with hippocampal PET SUVR.

Findings were unchanged after excluding one outlier with markedly elevated hippocampal tau (SUVR = 2.93; next highest < 1.85) and after excluding two participants classified as AD who were Aβ− .

## 4. Discussion

In a cohort of 403 older adults from the ADNI study, we examined the association between depressive symptoms, measured with the GDS, and hippocampal tau deposition measured with flortaucipir PET. An inverse correlation between level of depression and hippocampal tau was observed in participants without AD pathology, defined by the absence of Aβ. In contrast, in Aβ+ individuals, there was a trend level positive correlation between hippocampal tau and depression; these two correlations differed significantly from each other, and a significant interaction between depression and Aβ+ status was confirmed in a unified regression model. Results in Aβ+ individuals are expected and align with decades of neuroimaging and autopsy studies indicating that medial temporal hyperphosphorylated tau deposition in tangles is closely linked to neurodegeneration and neuropsychiatric dysfunction in individuals with AD pathology (Ossenkoppele et al., 2016; Bejanin et al., 2017).

Results in Aβ− individuals of an of an inverse association between depression and hippocampal tau (greater depression with less tau) are at first glance unexpected given the clearly pathologic role of tau in AD. We considered whether hippocampal atrophy, which is associated with depression, (Videbech and Ravnkilde, 2004) could artifactually lower apparent PET signal. However, results remained significant after adjusting for hippocampal volume, as well as for other variables (age and cognition). Current results therefore indicate that PET-measured medial temporal tau has different meaning and associations in individuals with versus without AD, and in the absence of AD pathology, higher tau levels are not pathological, at least not with respect to depression. These results are concordant with our recent prior study using a 2nd generation tau PET tracer (Glaubitz et al., 2025), and with results from others showing that medial temporal tau is linked to psychiatric symptoms *only* in Aβ+ individuals with confirmed AD pathology (Talmasov et al., 2025; Li et al., 2024), with hints of reversed relation in individuals without AD pathology (Gonzales et al., 2021; Naude et al., 2024), as explicitly demonstrated here.

These results suggest that in the absence of visible AD pathology, tau PET signal in hippocampus reflects a normal physiologic substance or process that is reduced in the setting of depression. We hypothesize that this process may be hippocampal neurogenesis, which is known to occur in adult humans, to be reduced in depression (Eisch and Petrik, 2012; Sahay and Hen, 2007) and to require tau phosphorylation as part of microtubule-mediated cell division. Normally-phosphorylated tau is present in dividing and newborn and neurons and has been used as a marker of neurogenesis in preclinical studies (Wang and Mandelkow, 2016; Hong et al., 2010). We propose that tau PET may be sensitive to this physiological level of phosphorylated tau in the hippocampus, and that abnormally low hippocampal tau PET signal in depressed, Aβ− individuals may reflect impaired adult neurogenesis, although this interpretation remains speculative. Given that attempts to image neurogenesis in vivo have been largely unsuccessful (Simard et al., 2025), there is a clear need for noninvasive biomarkers of this process, and future focused studies will be important to determine whether tau PET could fulfill this role.

## 5. Limitations

Our study has important limitations. The use of the GDS as a self-report measure of depressive symptoms, the lack of information on antidepressant use, and the exclusion of individuals with clinically diagnosed major depression in ADNI represent important limitations. In particular, the absence of participants with clinically significant depression restricts the range of depressive symptoms and limits the ability to generalize these findings to clinically depressed populations. Future studies should incorporate more comprehensive psychiatric characterization, including formal clinician diagnosis of depression and a broader range of symptom severity, with concurrent AD biomarker assessment to capture both psychiatric and cognitive dimensions of brain function rather than focusing on either domain in isolation. Use of the first generation tau tracer, flortaucipir, is a limitation due to off-target binding to choroid plexus and extracerebral structures, which makes accurate quantification of tau, particularly in medial temporal lobe, challenging (Leuzy et al., 2019). Mitigating against this, we previously found similar results using a second generation tau tracer with less off-target binding (Glaubitz et al., 2025). Most critically, it remains uncertain whether tau PET tracers bind normally phosphorylated tau outside neurofibrillary tangles, and, if so, whether PET has sufficient sensitivity to detect it given the low intracellular levels of phosphorylated tau within dividing or newborn neurons and the small number of such neurons in the adult hippocampus.

## 6. Conclusion

In the absence of AD pathology, greater depression severity is associated with lower PET-measured hippocampal tau. Because impaired hippocampal neurogenesis is considered a key pathophysiology of depression, and tau is a normal component of cell division including neurogenesis, this replicated inverse relationship may reflect reduced hippocampal neurogenesis in depression and suggests that tau PET could provide a window into this process in humans. Further studies are needed to test this hypothesis.

## Figures and Tables

**Table 1:** Participant characteristics.

Characteristic	Total (*N* = 403)	Aβ+ (*n* = 151)	Aβ− (*n* = 252)	p-value

Age in years (SD, range)	73.2 (7.3, 55–95)	75.6 (7.2, 55–94)	71.8 (7.1, 55–95)	<0.001
Female % (n)	53.8 % (217)	54.3 % (82)	53.6 % (135)	0.97
Diagnosis (CN/MCI/AD) %	64.3 / 33.0 / 2.7	51.7 / 42.4 / 6.0	72.2 / 27.4 / 0.8	<0.001
GDS score (SD, range)	1.23 (1.57, 0–10)	1.46 (1.56, 0–8)	1.10 (1.56, 0–10)	0.026
Hippocampal tau SUVR (SD, range)	1.20 (0.19, 0.67–2.93)	1.28 (0.24, 0.74–2.93)	1.15 (0.14, 0.67–1.79)	<0.001
Tau positive* % (n)	16.6 % (67)	40.4 % (61)	2.4 % (6)	<0.001

* Tau positivity was defined as SUVR > 1.3 within a temporal meta region.

Abbreviations: CN = Cognitively Normal, MCI = Mild Cognitive Impairment; SD = standard deviation, Aβ = amyloid-βeta, GDS = Geriatric Depression Scale, SUVR = Standardized Uptake Value Ratio.

## References

[R1] Herrman H, Patel V, Kieling C, , 2022. Time for united action on depression: a Lancet–World Psychiatric Association Commission. The Lancet 399 (10328), 957–1022. 10.1016/S0140-6736(21)02141-3 35180424

[R2] Eisch AJ, Petrik D, 2012. Depression and hippocampal neurogenesis: a road to remission? Science 338 (6103), 72–75. 10.1126/science.1222941. 23042885 PMC3756889

[R3] Eriksson PS, Perfilieva E, Björk-Eriksson T, , 1998. Neurogenesis in the adult human hippocampus. Nat. Med. 4 (11), 1313–1317. 10.1038/3305. 9809557

[R4] Sahay A, Hen R, 2007. Adult hippocampal neurogenesis in depression. Nat. Neurosci. 10 (9), 1110–1115. 10.1038/nn1969. 17726477

[R5] Simard S, Matosin N, Mechawar N, 2025. Adult hippocampal neurogenesis in the Human brain: updates, challenges, and perspectives. The Neuroscientist 0 (0). 10.1177/10738584241252581, 10738584241252581. 38757781

[R6] Glaubitz E, Wang XH, Xi K, , 2025. PET-measured tau deposition in emotion-related brain regions is differentially associated with depressive symptoms in individuals with versus without Alzheimer’s disease pathology. Behav. Brain Res, 115889 41176285 10.1016/j.bbr.2025.115889 PMC13179103

[R7] Wang Y, Mandelkow E, 2016. Tau in physiology and pathology. Nature Reviews Neuroscience 17 (1), 22–35. 10.1038/nrn.2015.1. 26631930

[R8] Hong XP, Peng CX, Wei W, 2010. Essential role of tau phosphorylation in adult hippocampal neurogenesis. Hippocampus 20 (12), 1339–1349. 10.1002/hipo.20712. 19816983

[R9] Ossenkoppele R, Schonhaut DR, Schöll M, , 2016. Tau PET patterns mirror clinical and neuroanatomical variability in Alzheimer’s disease. Brain 139 (5), 1551–1567. 10.1093/brain/aww027. 26962052 PMC5006248

[R10] Bejanin A, Schonhaut DR, La Joie R, , 2017. Tau pathology and neurodegeneration contribute to cognitive impairment in Alzheimer’s disease. Brain 140 (12), 3286–3300. 10.1093/brain/awx243. 29053874 PMC5841139

[R11] Ownby RL, Crocco E, Acevedo A, , 2006. Depression and risk for Alzheimer Disease: systematic review, Meta-analysis, and metaregression analysis. Arch. Gen. Psychiatry 63 (5), 530–538. 10.1001/archpsyc.63.5.530. 16651510 PMC3530614

[R12] Saczynski JS, Beiser A, Seshadri S, , 2010. Depressive symptoms and risk of dementia. Neurology 75 (1), 35–41. 10.1212/WNL.0b013e3181e62138. 20603483 PMC2906404

[R13] Green RC, Cupples LA, Kurz A, , 2003. Depression as a risk factor for Alzheimer Disease: the MIRAGE study. Arch. Neurol. 60 (5), 753–759. 10.1001/archneur.60.5.753. 12756140

[R14] Videbech P, Ravnkilde B, 2004. Hippocampal volume and depression: a meta-analysis of MRI studies. Am. J. Psychiatry 161 (11), 1957–1966. 10.1176/appi.ajp.161.11.1957. 15514393

[R15] Talmasov D, Johnson AS, Brown PJ, , 2025. Depressive symptoms correlate with tau accumulation rates in amyloid positive adults. Am. J. Geriatr. Psychiatry. 10.1016/j.jagp.2025.04.207 PMC12782759 40280817

[R16] Li JS, Tun SM, Ficek-Tani B, , 2024. Medial amygdalar tau is associated with mood symptoms in preclinical Alzheimer’s Disease. Biolog. Psychiatry: Cognit. Neurosci. Neuroimag. 9 (12), 1301–1311. 10.1016/j.bpsc.2024.07.012 PMC11625605 39059466

[R17] Gonzales MM, Samra J, O’Donnell A, , 2021. Association of midlife depressive symptoms with regional amyloid-β and tau in the Framingham Heart Study. J. Alzheimers Dis. 82 (1), 249–260. 10.3233/jad-210232. 34024836 PMC8900661

[R18] Naude J, Wang M, Leon R, , 2024. Tau-PET in early cortical Alzheimer brain regions in relation to mild behavioral impairment in older adults with either normal cognition or mild cognitive impairment. Neurobiol. Aging 138, 19–27. 38490074 10.1016/j.neurobiolaging.2024.02.006

[R19] Leuzy A, Chiotis K, Lemoine L, , 2019. Tau PET imaging in neurodegenerative tauopathies—Still a challenge. Mol. Psychiatry 24 (8), 1112–1134. 10.1038/s41380-018-0342-8. 30635637 PMC6756230

